# Anterior cervical discectomy and fusion surgery versus total disc replacement: A comparative study with minimum of 10-year follow-up

**DOI:** 10.1038/s41598-017-16670-1

**Published:** 2017-11-27

**Authors:** Si-Dong Yang, Yan-Bo Zhu, Suo-Zhou Yan, Jun Di, Da-Long Yang, Wen-Yuan Ding

**Affiliations:** 1 Department of Spinal Surgery, The Third Hospital of Hebei Medical University, Shijiazhuang, 050051 China; 2 Laboratory of Molecular Biology, Center of Experimental Animals, Hebei Medical University, Shijiazhuang, 050017 China; 3 Department of Orthopaedic Surgery, The Third Hospital of Hebei Medical University, Shijiazhuang, 050051 China; 4 Hebei Provincial Key Laboratory of Orthopedic Biomechanics, Shijiazhuang, 050051 China

## Abstract

Based on long-term follow-ups, this study was designed to investigate the incidence and risk factors for postoperative adjacent segment degeneration (ASD) after anterior cervical discectomy and fusion (ACDF) or total disc replacement (TDR) in treating cervical degenerative diseases. Between January 2000 and December 2005, 108 cases undergoing ACDF and 78 undergoing TDR, were enrolled into this study. All medical records were retrospectively collected. Every patient was followed up at least 10 years. Outcome assessment included visual analogue scale (VAS) score, Neck Disability Index (NDI) score, Japanese Orthopaedic Association (JOA) score, and radiographic parameters. Consequently, thirty-eight (35.2%) of 108 cases suffered from ASD in ACDF group, and 26 (33.3%) of 78 cases in TDR group. There was no statistical difference between the two groups regarding ASD incidence, VAS/NDI/JOA score, recovery rate. Logistic regression analysis showed that age (OR = 2.86, 95% CI, 1.58–4.14) and preoperative segmental lordosis (OR = 1.90, 95% CI, 1.05–3.20) were risk factors associated with increased odds of ASD regardless of surgical procedures. On the other hand, preoperative overall lordosis (OR = 0.54, 95% CI, 0.26–0.82) was most likely protective. In conclusion, advanced age and preoperative segmental lordosis were identified as risk factors for postoperative ASD, while preoperative overall lordosis proves to be a protective factor.

## Introduction

Anterior cervical discectomy and fusion (ACDF) which has been considered as the “gold standard” treatment of cervical degenerative diseases was founded by Smith-Robinson and Cloward inthe 1950s^1^. But in recent years, radiographic and clinical studies have shown that as time went by, the segements adjacent to the fused spinal segments occasionally degenerated or became unstable^2–4^.

ACDF surgery has changed the original mechanical behavior of the spine at the expense of the activity of the fusion segment; and in theory it may lead to the changes of adjacent vertebral stress distribution and the movement patterns, resulting in biomechanical changes including stress concentration of adjacent segments, compensatory increase in activity, and even instability^5–7^. However, the correlation between so-called “adjacent segment degeneration” and ACDF surgery still needs theoretical and experimental data to support. It is unclear whether ASD after ACDF surgery occurs due to segmental fusion, or it is just the normal physiological degeneration of the spine.

Nowadays, cervical total disc replacement (TDR) is a major non-fusion surgical method. Its designed concept is to retain as much as possible the intervertebral disc height and segmental activity, so as to reduce accelerated adjacent segment degeneration (ASD) that may be caused by ACDF surgery. Its short-term clinical results have been well demonstrated^3,8–16^, but the studies reporting long-term curative effect are still few^17^.

Thus, this study was designed to investigate the incidence and risk factors for postoperative ASD after ACDF or TDR surgery, and compare the clinical effects in treating cervical degenerative diseases, based on a long-term follow-up with a minimum of 10 years.

## Patients and Methods

### Statement

This study was approved by the local Ethics Committee of The Third Hospital of Hebei Medical University (Approval No. K2017–003–01). Informed consent was obtained from all participants and/or their legal guardian/s. The methods were carried out in accordance with the relevant guidelines and regulations.

### Patient selection

Case selection had strict criteria as follows. Inclusion criteria are (1) single-level, radiculopathic; (2) cervical disc herniation; (3) degenerative cervical spinal stenosis; (4) conservative treatment for at least three months; (5) adult patients only; (6) no degeneration existing in the adjacent disc. Exclusion criteria are (1) severe facet joint degeneration (bridging osteophytes, intervertebral disc height loss > 50%, intervertebral activity < 2°); (2) developmental cervical stenosis; (3) ossification of posterior longitudinal ligament; (4) obviously unstable cervical spine with angular displacement > 2° or vertical displacement > 2 mm; (5) osteoporosis, or with spinal compression fractures; (6) cervical abnormalities; (7) cervical cancer; (8) cervical infection; (9) ankylosing spondylitis; (10) a history of cervical spine surgery.

It should be noted here that both the two groups of patients were not randomly selected; all included patients were suitable for TDR surgery or ACDF surgery. But taking account of the ethical issues and economic capacity of the patients, the final choice of selecting an operation was made by the patients themselves after a complete explanation to them before surgery.

### Surgical procedures

**Figure 1:**
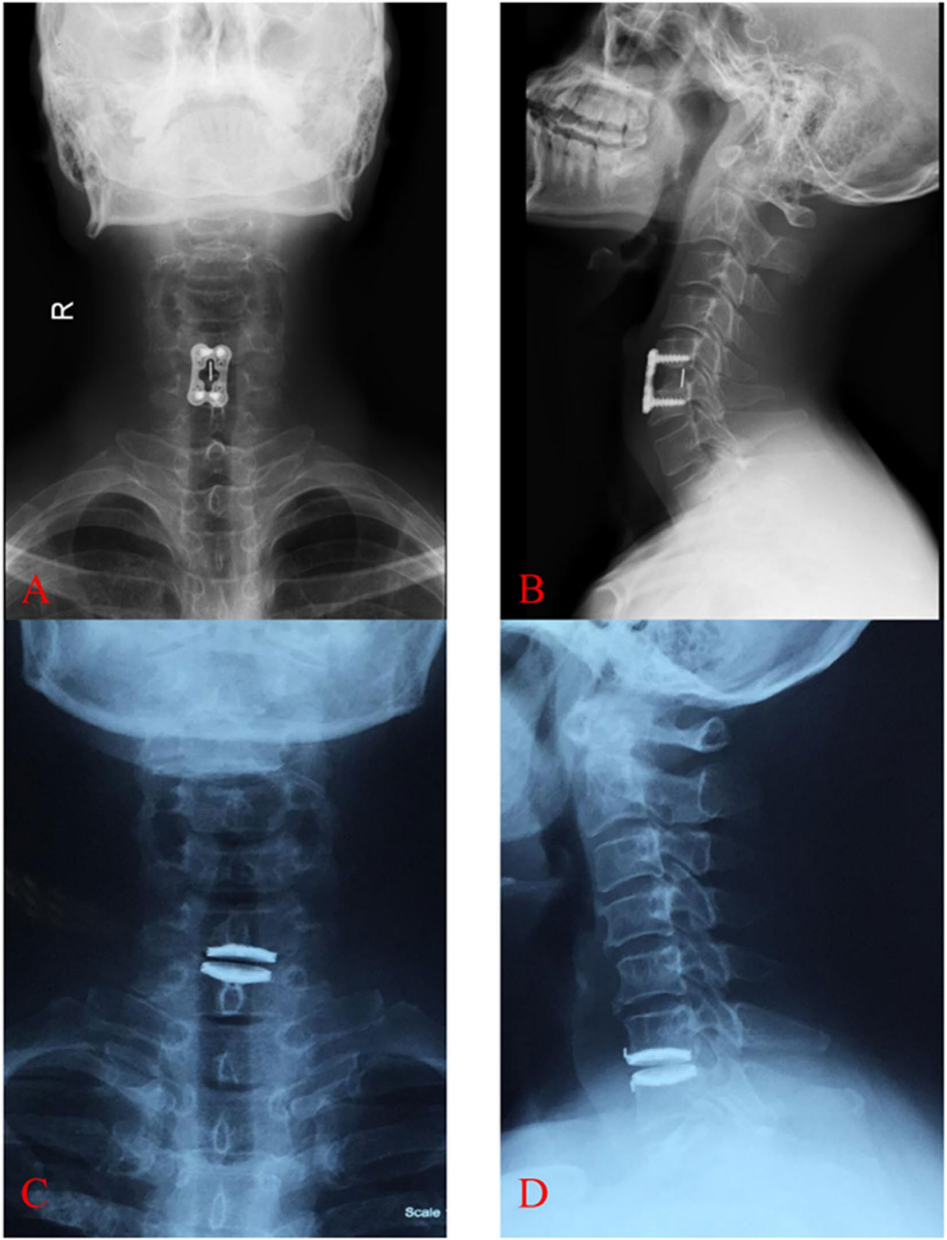
X-ray images of ACDF and TDR surgery in treating cervical degenerative diseases. (**A,B**) The Syncage-C (Synthes company) or PEEK-Cage (Depuy company) with local bone was implanted into intervertebral space in ACDF group. (**C,D**) For TDR group, the Bryan Cervical Disc Prosthesis (Medtronic Sofamor Danek, Inc, Memphis, TN) was implanted into intervertebral space after accurate measurement. ACDF, anterior cervical discectomy and fusion; TDR, total disc replacement.

Surgery was performed with conventional technique as follows. Briefly, a standard right-sided anterior approach was performed. The symptomatic disc was removed, and then the posterior longitudinal ligament (PLL) was removed. The Syncage-C (Synthes company) or PEEK-Cage (Depuy company) with local bone was implanted into intervertebral space in ACDF group, as shown in Fig. 1A,B. For TDR group, the Bryan Cervical Disc Prosthesis (Medtronic Sofamor Danek, Inc, Memphis, TN) was implanted after accurate measurement, as shown in Fig. 1C,D.

### Radiographic measurements

Radiographic parameters were collected by screening neutral and dynamic flexion-extension lateral radiographs, including cervical lordosis, operated segmental height, C2-7 range of motion (ROM), operated segmental ROM, upper segmental ROM and lower segmental ROM, upper segmental height and lower segmental height. Neutral-position and dynamic flexion-extension lateral radiographs during each follow-up examination were evaluated with the PACS software and a PACS workstation (Centricity 2.0, General Electrics Medical Systems, Milwaukee, WI).

ASD was assessed through lateral X-ray film and MRI T2-weighted images. The Kellgren X-ray cervical vertebra degeneration system was a method considering the cervical degeneration just like the anterior vertebral osteophyte, disc height collapse, endplate sclerosis and anterior or posterior slip. The MRI manifestation of ASD was newly formation of intervertebral disc herniation and decreased signal intensity on MRI images using Miyazaki classification^18^. All radiological outcomes were reviewed by an independent spine surgeon and a radiologist, who were unaware of the treatment details. At last follow-up, the cases whose X-ray and (or) MRI appear ASD performance were included in ASD group, otherwise, included in non-ASD group.

### Outcome assessment

All patients were required to return for follow-up. Clinical and radiological evaluation were performed at 1 week, 3 months, 1 year, postoperatively, and last follow-up (more than 10 years). Clinical effectiveness was evaluated by visual analogue scale (VAS) score, Japanese Orthopaedic Association (JOA) score (17 points system, 1994 revised edition)^19,20^ and Neck Disability Index (NDI) score. The recovery rate (RR) of JOA score was calculated according to the following formula: RR = (postoperative scores – preoperative scores)/(17 - preoperative scores) *100%.

### Statistical analysis

Statistical analyses were performed using SPSS for Windows, version 18.0 (IBM SPSS Inc., New York, USA). Data are presented as Mean ± SD (standard deviation) for measurement data. Comparisons of VAS, JOA, NDI score, and preoperative radiographic parameters between ACDF group and TDR group were determined by Student t-test. For count data, it was presented as percentage, and chi-square test was used for data analysis. Non-conditional binary logistic regression model was used to explore the associated risk factors of ASD in patients after surgery. P values less than 0.05 were regarded as significant with two-tailed tests.

## Results

### Baseline data

This study was carried out between January 2000 and December 2005. As shown in Table 1, a total of 186 patients underwent ACDF surgery (108 cases), or Bryan (Medtronic Sofamor DanekInc, USA) TDR surgery (78 cases). The age is 50 ± 18 years in ACDF group, and 52 ± 19 years in TDR group. All cases have completed regular follow-ups of at least 10 years. The baseline data from two groups showed no significant difference regarding age, sex, and history of chronic diseases including diabetes mellitus, heart diseases, and high blood pressure. All operations were performed by the same surgeon (WYD).

**Table 1:** Baseline data of the study population.

Groups	Sex		Age (yrs)	Chronic diseases		
	Male	Female		DM	HD	HBP
ACDF(n = 108)	51	57	50 ± 18	21	10	23
TDR(n = 78)^abc^	38	40	52 ± 19	14	7	16

Note: ACDF, anterior cervical discectomy and fusion; TDR, total disc replacement; DM, diabetes mellitus; HD, heart disease; HBP, high blood pressure.

^a^P = 0.96, compared to ACDF group regarding sex distribution.

^b^P = 0.47, compared to ACDF group regarding age.

^c^P = 0.995, compared to ACDF group regarding the distribution of chronic diseases.

### ASD incidence

Two groups of patients successfully completed surgery without major complications. At last follow-up, it was found that 38 (35.2%) of 108 cases suffered from ASD in ACDF group, and 26 (33.3%) of 78 cases in TDR group. There was no statistical difference between the two groups regarding ASD incidence. We compared ASD group with non-ASD group and found that sex, disease duration, surgical approach and follow-up period had no significant differences (all P > 0.05). But the patients in ASD group (56 ± 12 years) are older than those in non-ASD group (50 ± 11 years) (P < 0.001).

### VAS/JOA/NDI scores and RR

At last follow-up, VAS/JOA/NDI scores both in ACDF and TDR groups achieved significant improvement compared with the preoperative scores, as shown in Tables 2–4. However, there were no significant differences regarding VAS/NDI/JOA scores between ACDF group and TDR group. As well, there was no statistical difference regarding RR between ACDF group (RR = 75.56%) and TDR group (RR = 81.58%).

**Table 2:** Comparison of VAS score between ACDF group and TDR group by Student t-test.

Group	Preoperative	1 wk	3 months	1 yr	last follow-up
ACDF	6.5 ± 2.5	4.0 ± 2.2	2.5 ± 1.1	2.0 ± 1.5	1.9 ± 1.0*
7.2 ± 2.3	4.2 ± 2.5	2.0 ± 0.8	1.8 ± 1.2	1.7 ± 1.1*	7.2 ± 2.3
P-value	0.651	0.752	0.689	0.805	0.832

Note: ACDF, anterior cervical discectomy and fusion; TDR, total disc replacement.

*P < 0.05, compared with preoperative data.

**Table 3:** Comparison of JOA score between ACDF group and TDR group by Student t-test.

Group	Preoperative	1 wk	3 months	1 yr	last follow-up
ACDF	9.4 ± 1.3	13.8 ± 1.5	15.3 ± 1.1	15.1 ± 1.0	15.5 ± 1.0*
TDR	10.2 ± 1.3	13.1 ± 1.0	15.0 ± 1.5	15.0 ± 1.1	15.0 ± 1.6*
P-value 0.590	0.590	0.722	0.856	0.867	0.755

Note: ACDF, anterior cervical discectomy and fusion; TDR, total disc replacement.

*P < 0.05, compared with preoperative data.

**Table 4:** Comparison of NDI score between ACDF group and TDR group by Student t-test.

Group	Preoperative	1 wk	3 months	1 yr	last follow-up
ACDF	35.6 ± 13.5	22.4 ± 7.3	14.4 ± 5.7	15.0 ± 4.5	14.2 ± 3.8*
TDR	34.3 ± 12.0	20.3 ± 8.5	15.5 ± 6.1	14.0 ± 5.4	13.5 ± 3.1*
P-value	0.671	0.463	0.774	0.827	0.827

Note: ACDF, anterior cervical discectomy and fusion; TDR, total disc replacement.

*P < 0.001, compared with preoperative data.

### Preoperative radiographic parameters

As shown in Table 5, preoperative overall lordosis is 12.6 ± 1.1 in ASD group while it is 15.3 ± 1.4 in Non-ASD group. Thus, preoperative overall lordosis in ASD group is smaller than that in Non-ASD group (P < 0.001). Preoperative segmental lordosis is 2.8 ± 4.0 in ASD group while it is 1.1 ± 3.8 in Non-ASD group, with significant difference (P = 0.047). There were no statistical differences regarding ROM, segment height, or segmental activity between ASD group and Non-ASD group (all P > 0.05).

**Table 5:** Comparison of preoperative radiographic parameters between ASD group and Non-ASD group by Student t-test.

Group	OL	SL	C-ROM	U-ROM	L-ROM	USH	LSH	SA
ASD	12.6 ± 1.1	2.8 ± 4.0	42.5 ± 5.6	8.3 ± 1.9	7.8 ± 1.1	12.6 ± 1.8	12.6 ± 1.6	9.25 ± 3.5
Non-ASD	15.3 ± 1.4	1.1 ± 3.8	43.9 ± 6.5	10.6 ± 1.5	8.4 ± 1.2	13.5 ± 2.0	13.3 ± 1.4	9.59 ± 3.2
P-value	001	0.047	0.558	0.436	0.621	0.535	0.677	0.832

Note: OL, overall lordosis; SL, segmental lordosis; C-ROM, C2-C7 ROM; U-ROM, Upper Segment ROM; L-ROM, Lower Segment ROM; USH, Upper Segment Height; LSH, Lower Segment Height; SA, segmental activity.

### Logistic regression analysis

All patients were divided into three age groups according to WHO classification (~44; 45~59; 60~). As shown in Table 6, logistic regression analysis showed that the regression equation was presented as follows, logit P = −3.28 + 1.05*X1 − 0.62*X3 + 0.64*X4 (X1 = age, OR = 2.86, 95% CI, 1.58–4.14; × 3 = preoperative overall lordosis, OR = 0.54, 95% CI, 0.26–0.82; × 4 = preoperative segmental lordosis, OR = 1.90, 95% CI, 1.05–3.20). Herein, ‘logit P’ is the likelihood of developing an ASD after a surgery, regardless of the technical procedures used.

**Table 6:** Binary logistic regression analysis of postoperative ASD.

No.	Items	B	Exp(B)	*p*-value	95% CI for Exp(B)
X1	Age	1.05	2.86	<0.001	(1.58, 4.14)
X2	SM	0.76	2.14	0.359	(0.51, 10.25)
X3	OL	−0.62	0.54	0.014	(0.26, 0.82)
X4	SL	0.64	1.90	0.003	(1.05, 3.20)
X0	Constant	−3.28	0.04	<0.001	—

Note: SM, surgical method; OL, overall lordosis; SL, segmental lordosis.

## Discussion

In our study, postoperative neurological function scores of both ACDF group and TDR group were significantly different from preoperative scores respectively, and there was no significant difference between the two groups at different timepoints. It showed that the postoperative clinical results, involving significant improvement in neurological function and quality of life were satisfactory when compared with preoperative status, regardless of surgical procedures, ACDF or TDR. Reported literature (randomized controlled clinical studies) had shown that the short-term effect of TDR surgery was not inferior to ACDF surgery^21–23^. These results were similar to those of our study. The same surgery point of the two surgical ways above was the operation of thoroughly complete decompression in the pathological level. It might indicate that recovery of neurological function was primarily related to whether intraoperative decompression was thorough or not, not the selection of surgical methods.

Some reported literature indicated that cervical sagittal curvature had an important impact on neck pain, spinal cord compression and ASD^24,25^. Pickett *et al*.^26^ and Johnson *et al*.^27^ reported that Bryan TDR surgery might bring about deformity of segmental kyphosis. In this study, we found that the ROM recovered to the preoperative value during the follow-up period after Bryan TDR surgery. Meanwhile, the treated segment ultimately showed preservation of movement when compared with the preoperative levels. Postoperative overall lordosis angle after ACDF surgery was basically consistent with the preoperative status. As compared with ACDF, Bryan TDR surgery had a better effect on recovery and reconstruction of overall lordosis angle. However, the Bryan TDR surgery was non-fusion surgery without titanium plate fixation. How to avoid the kyphosis that the above scholars reported, and how to achieve overall lordosis improvement? We should pay more attention to the following aspects: (1) to keep neutral position and avoid hyper-extension during operation; (2) to thoroughly remove the osteophytes at anterior lower edge of the upper vertebral body, and to avoid improper intervertebral height resulting in excessive lordosis; (3) to determine the exact prosthesis model through accurately measuring the size of vertebral body in X-ray images; (4) to be sure that insertion direction of prosthesis was parallel to the intervertebral space; (5) excessive scraping on cartilage endplate should be avoided, especially near to the dorsal side.

Cervical local and overall activity was critical for both ACDF surgery and TDR surgery. In this study, the difference of preoperative overall activity between the two groups was not statistically significant. At last follow-up, overall activity in TDR group decreased average 1.8° compared with preoperative data, while that in ACDF group decreased average 4.0° compared with preoperative data. It indicated that TDR surgery had significant effect of maintaining cervical overall activity. The surgery has been widely used in clinical practice since 2000 and has received good clinical efficacy. Quan *et al*.^28^ followed up the patients who had underwent Bryan TDR surgery for more than 8 years, 78% of the patients still maintained a good activity. In the present study, we have found that TDR has the role of maintaining overall and segmental activity significantly, which is consistent with the previous report.

In this study, we measured intervertebral disc height and mobility of adjacent segments both before and after surgery in ACDF and TDR group. Intervertebral disc height and ROM were currently the standard evaluation method in ASD applied by the majority of authors. The results showed that intervertebral disc height and ROM of adjacent segments had no significant differences before and after surgery, regardless of TDR group or ACDF group. Sasso *et al*.^29^ followed up 22 patients who underwent TDR surgery and found that the upper adjacent vertebral activity was about 10° and the lower was about 15^°^ two years after the surgery. They concluded with prolonged follow-up period that the vertebral activity of adjacent segments increased after ACDF surgery, which was consistent with the findings in our study.

Long-term follow-up interview after ACDF surgery indicated that there were many complications, such as subsequent instability, loss of physical activity, ASD and so on. ASD pathological changes mainly included cervical spondylosis changes, such as cervical osteophyte formation around the vertebral body, disc space narrowing, vertebral slippage, disc herniation, hypertrophy and ossification of the yellow ligament in X-ray and/or CT/MRI images. All of the proliferative and degenerative performance will inevitably lead to degenerative cervical stenosis of the fused and adjacent segments, resulting in spinal cord injury and a corresponding neurological symptoms^30^. ASD has an adverse impact on the long-term effect on the recovery for patients undergoing ACDF surgery, which has become a major anterior cervical complication.

In the prior univariate analysis, it was found that the patients in ASD group (56 ± 12 years) are older than those in non-ASD group (50 ± 11 years) (P < 0.001). In the logistic regression analysis, all patients in this study were divided into three age groups according to WHO classification (~44; 45~59; 60~), and advanced age was identified as a risk factor of postoperative ASD. We can identify advanced age as a risk factor, but we cannot set the range of age. Cervical lordosis is the biomechanically ideal alignment, but it refers to the overall lordosis. In this study, high-level segmental lordosis was identified as a risk factor of postoperative ASD, whereas high-level overall lordosis was observed to act as a protective factor.

This study goes along with some limitations. First, ACDF surgery and TDR surgery were not randomly selected for the patients, because both two procedures were suitable for patients and the final surgical option was determined by the patients mainly depending on their medical expense. Second, the non-randomized character of the study design was bound to cause some selection bias, even though the baseline and demographic data in the two groups had no statistically significant differences. In addition, the small sample size was also a limitation in this study. This study only looked at radiographic ASD, which is another limitation.

Osteoporosis may lead to the deformation of the cervical vertebra and cause the stenosis of the vertebral canal, which is a potential risk factor for promoting the degeneration of cervical vertebra and developing into cervical spondylosis^31^. As a retrospective study, osteoporosis cannot be checked for in all patients; thus, only some patients were checked for osteoporosis after being admitted to our hospital. We only can judge the osteoporosis of the rest patients by screening the existed X-ray photographs. It is really another limitation in this study. At last, no recording of other complications including reoperation, symptom recurrence, is also a limitation in this study.

In summary, both ACDF surgery and TDR surgery for cervical single-level degenerative diseases have achieved satisfying clinical results. Postoperative ASD incidence in the two groups has no significant difference. Advanced age and high-level preoperative segmental lordosis were identified as risk factors for postoperative ASD, while high-level preoperative overall lordosis may act as a protective factor.
